# Economic Costs of Cardiovascular Diseases in Poland Estimates for 2015–2017 Years

**DOI:** 10.3389/fphar.2020.01231

**Published:** 2020-09-08

**Authors:** Aneta Mela, Elżbieta Rdzanek, Łukasz A. Poniatowski, Janusz Jaroszyński, Marzena Furtak-Niczyporuk, Małgorzata Gałązka-Sobotka, Dominik Olejniczak, Maciej Niewada, Anna Staniszewska

**Affiliations:** ^1^ Department of Experimental and Clinical Pharmacology, Centre for Preclinical Research and Technology (CePT), Medical University of Warsaw, Warsaw, Poland; ^2^ Department of Neurosurgery, Maria Skłodowska-Curie Memorial Cancer Center and Institute of Oncology, Warsaw, Poland; ^3^ Chair of Administrative Procedure, Faculty of Law and Administration, Maria Curie-Skłodowska University of Lublin, Lublin, Poland; ^4^ Department of Public Health, Faculty of Medicine, Medical University of Lublin, Lublin, Poland; ^5^ Department of Management and Marketing, Faculty of Economics and Management, Łazarski University, Warsaw, Poland; ^6^ Institute of Management in Health Care, Łazarski University, Warsaw, Poland; ^7^ Department of Public Health, Faculty of Health Sciences, Medical University of Warsaw, Warsaw, Poland

**Keywords:** cardiovascular diseases, direct cost, indirect costs, economic burden, work lost, cost of illness, health economics

## Abstract

**Background:**

Cardiovascular diseases are associated with growing public and private expenditure on healthcare regardless geographic region. Therefore, it is necessary to accurately estimate the overall societal costs—both direct and indirect expenses from the perspective of patients, caregivers and employers.

**Research Design:**

The aim of this paper is to determine the direct and indirect costs related to cardiovascular diseases in Poland from 2015 to 2017. All costs are estimated based on data available in the public domain and obtained from the major Polish institutions. Indirect costs were calculated using a modified human capital approach.

**Results:**

The financial burden of cardiovascular diseases in Poland is significant. This study revealed that total costs (direct and indirect) of cardiovascular diseases, for 2015–2017, range from 34.9 bn PLN (8.2 bn EUR) to over 40.9 bn PLN (9.6 bn EUR). Total direct cost and indirect costs were approximately 6.1 bn PLN (1.4 bn EUR) (16%) and 31.3 bn PLN (7.3 bn EUR) (84%), respectively.

**Conclusion:**

Collectively, the estimated direct and indirect cost of cardiovascular diseases provide a useful input for economic impact assessments of public health programs and health technology analyses.

## Introduction

As was stated by World Health Organization (WHO), the four predominant types of NCD are cardiovascular diseases (CVD) including acute coronary syndrome (ACS) and stroke, cancer and other neoplasms, chronic respiratory diseases including chronic obstructed pulmonary disease (COPD) and asthma as well as diabetes mellitus (DM) (World Health Organization. (WHO), 2014; Siqueira et al., 2017). Among enlisted entities the CVD were responsible for the nearly ~37% of deaths below age of 70 years (World Health Organization. (WHO), 2014). According to the American Heart Association (AHA) statement the CVD constitute primary cause of mortality in the United States (US) affecting in this case 1/3 of adult population with the projection to increase by 2030 to 40.5% of the population (Calafiero and Jané-Llopis, 2011; Williams et al., 2018). The data from the European Society of Cardiology (ESC) and the Fourth Joint Task Force indicate that CVD, around the year 2000, was the direct cause of at least 4 million deaths in Europe; including 1.9 million in the European Union (EU) causing more than 50% of all deaths across Europe (Heidenreich et al., 2011).

Collectively, taking into consideration vast epidemiological range, it is not surprising that the CVD due to their associated long-term detrimental outcomes and equivalent socioeconomic constitutes substantial global economic burden regardless geographic region (Mensah and Brown, 2007). The contributing factors include, but are not limited to, direct costs including hospitalizations, physician visits, diagnostics, rehabilitation services, and drugs. As well as, indirect costs associated with mortality and morbidity including loss of productivity due to premature mortality, reduced employment, un-employment, and short or long-term disability (Graham et al., 2007). The estimated 2010 costs of CVD globally covered about 863 bn USD whereas it is predicted to rise by 22% to 1.044 bn USD by 2030 (Kuehn, 2013). In the US CVD-related direct medical costs and indirect costs were 273 bn USD and 172 bn USD, respectively with the prediction to rise to 276 bn USD by 2030 considering indirect costs (Calafiero and Jané-Llopis, 2011). Regarding the data from EU the estimated costs of CVD in 2003 was 169 bn EUR (Tarride et al., 2009).

Taking into account all mentioned clinico-epidemiological aspects it become essential to accurately estimate the overall societal costs of CVD, both direct and indirect from points of view including patients, caregivers and employers (World Economic Forum, 2011). Precisely, this concern should focus on the impact of employee health on workplace productivity and its potential loss which is related with specific health factors conditions especially among CVD (Leal et al., 2006; Song et al., 2015). Therefore, the management and prevention of CVD should become first health priority imposed on governments and politics regardless geographic area (Milani and Lavie, 2009).

According to the report, Organization for Economic Co-operation and Development (OECD) and European Observatory on Health Systems and Policies (EOHSP), in collaboration with the European Commission in 2017, in Poland the CVD constitute leading cause of death is responsible for 50 and 40% of all deaths among women and men (Boles et al., 2004). At the same time, the probability of death due to CVD in the Polish population is about 60% higher than for the average EU resident (OECD/European Observatory on Health Systems and Policies, 2017). In recent decades Poland, as an Eastern Europe country experienced rapid socioeconomic transitions, which resulted in considerable changes in the distribution of leading causes of morbidity and mortality during that period (Gaziano et al., 2010). Analysis of the current population age structure in Poland indicate that the great part of the community are in the 25–70 age group with an observed peak at the 20–40 group which allows presumption despite many advances in prophylaxis and treatment of CVD that its prevalence will still increase in Poland regarding extension of lifespan (Gaziano et al., 2010; OECD/European Observatory on Health Systems and Policies, 2017). However, in recent years Poland has strengthened its hospital sector providing relatively effective and high quality care to cardiac patients. This is demonstrated by one of the lowest mortality rates from heart attacks among all EU countries (Boles et al., 2004). Better information and monitoring systems were also implemented from 2004 to 2005 covering the cardiovascular registries, including the Polish Registry of Acute Coronary Syndromes (PL-ACS) and the Polish National Registry of Cardiac Surgery Procedures (KROK) (Ludność; Islami et al., 2011). Therefore, in spite of this evidence on the real direct and indirect economic costs of the management of CVD in Poland is lacking. Hence, the accurate information and data regarding the costs attributable to the management of CVD to be covered by the society and payer in respective European countries including Poland seems to be necessary. There are few available literature reports which have estimated costs related with introduction of primary prevention programs or single CVD entity and procedure such as heart failure, hypertension and percutaneous coronary intervention (PCI) (Poloński et al., 2007; Czech et al., 2013; Sović et al., 2013; Suwalski et al., 2018). Therefore, we performed a comprehensive pharmacoeconomic analysis to estimate and determine trends of direct and indirect cost distribution related to all CVD entities covered by the list derived from the 10th version of the International Classification of Disease (ICD-10) including I00-I99 codes in Poland during the years 2015–2017.

The aim of this paper is to measure both direct and indirect costs of cardiovascular diseases (ICD-10: I00-I99) in Poland. The estimates were carried from the year 2015 to 2017. The period included in the analysis results directly from the availability of data enabling estimation of direct and indirect costs from publicly available data sources.

## Methods

### Study Population

We used data available in the public domain (National Health Fund (NHF), Central Statistical Office of Poland (CSO) as well as publicly unavailable data, obtained from the Social Insurance Institution (SII). The estimates were carried out for both direct and indirect costs. In the case of the first category of costs, public payer (NHF) expenses related to reimbursement of medicines and medical services (hospital treatment, outpatient specialist care, medical rehabilitation, health services contracted separately) were included. In terms of indirect costs (from a societal perspective), the costs of absenteeism, presenteeism and loss of production resulting from permanent or temporary inability to work or due to premature death have been taken into account. We did not include the costs of informal care in the analysis due to the lack of reliable data. The source data used to estimate the cost categories listed above are presented in **Table 1**. All estimates are expressed in both PLN (Polish zloty) and EUR (using the mean 2015–2017 exchange rate)^^1^^.

**Table 1:** Data sources.

Data category	Source	
Direct costs		
Medicinal products (drugs)	• Ministry of Health (MoH) Reimbursement list (Jan 2015 to Dec 2017) (https://www.gov.pl/web/zdrowie/) • NHF Economic and Financial Department’s statements (Jan 2015 to Dec 2017) (http://nfz.gov.pl/)	
Hospital treatment	• The value of NHF contracts (http://nfz.gov.pl/) concluded by individual NHF’ Provincial Departments in a given year o data divided into the service provider, type of service and the name of the contracted product • Publication: Rutkowski 2015	
Outpatient specialist care		
Medical rehabilitation		
Health services contracted separately		
Indirect costs		
Absenteeism	• Statistical Portal of SII (http://www.psz.zus.pl) o days of absence from work due to cardiovascular diseases (ICD-10: I00-I99) in 2015-2017	CSO data (http://www.stat.gov.pl/en) tabs: macroeconomic indicators: national accounts, labor market; population/survival tables: o GDP at current prices 2015–2017 o working population (end of period) 2015–2017 o general population in Poland—total population aged 18–59/64
Presenteeism	• Publications: Kotseva 2018 and POLKARD Report	
Permanent and temporary incapacity for work (pension)	• Statistical Portal of SII (http://www.psz.zus.pl) number of confirmed cases: inability to live independently, full disability to work and partial disability to work due cardiovascular diseases (ICD-10: I00-I99) in 2015-2017	
Mortality	• CSO data (http://www.stat.gov.pl/en), database: o demography – data on deaths by age and sex of the deceased and cause of death	

### Direct Costs

Direct costs related to the treatment of cardiovascular diseases in Poland from 2015 to 2017 are calculated based on data available in the public domain. The following categories of costs are taken into account: (i) cost of reimbursed drugs, (ii) cost of medical services used in cardiology in-hospital department and out-hospital consultation units, covered by the NHF. Other categories of costs (e.g. administration) were not included in the analysis due to the lack of data that would allow a reliable estimation.

#### Therapeutic Path of a Patient With CVD in Poland

A patient with a health problem will first report to a primary care physician, who plans and carries out medical care over the insured and coordinates the provision of services. If the patient’s condition requires further examination and treatment, he or she is referred to a specialist (e.g. cardiologist). During a visit to a specialist, the patient’s health is assessed and the further course of treatment determined based on the diagnostic tests performed. If a one-time consultation is sufficient, the specialist physician sends the patient back to the referring physician (primary care). In other cases, the patient is taken care of by a specialist clinic (including diagnostics). Patients receive tests, medicines and medical devices to the extent necessary to provide guaranteed services as part of outpatient specialist care. If the goal of treatment cannot be achieved on an outpatient basis, the patient is referred to the hospital. During hospital treatment, the service provider is obliged to provide the insured with necessary diagnostic tests and medicines related to the course of hospitalization (the use of which results from the reason for hospitalization).

#### Financing the Treatment of a Patient With CVD in Poland

An establishment that performs primary healthcare tasks receives an annual capitalization rate for each enrolled patient, regardless of the number of visits and diagnostic tests the patient receives in a given year. Such system does not distinguish a patient with cardiovascular disease in this analysis, therefore the cost of primary care has been omitted. Its inclusion would not be justified due to the inability to determine the number of visits or the type of diagnostic tests that the patient with CVD receives.

A visit to a specialist doctor and all diagnostic tests are financed as part of outpatient specialist care, while all services provided in the hospital are settled as part of hospital treatment. Hospital treatment includes all hospitalizations carried out as part of the Diagnosis Related Groups (DRG) system, including coronary artery bypass grafting, percutaneous valvuloplasty, coronary angioplasty with stent implantation, pacemaker implantation/replacement, treatment of congenital heart disease. Highly specialized procedures, such as e.g. heart transplantation or cardiological intervention in children under 18 years of age (including percutaneous leakage closure using closure kits) are financed as part of highly specialized services. The above cost categories have been included in our analysis.

#### Drugs Reimbursement

As a source of reimbursement data for drugs available within open pharmacies, the NHF Economic and Financial Department’s statements were used for the period from January 1, 2015 to December 31, 2017. We calculated expenditures on reimbursed drugs related to the cardiovascular system both from public payer and patient perspective. Identification of medicinal products was based on ATC (Anatomical classification) codes according to the EphMRA guidelines V2019 (class C – drugs related to the cardiovascular system). Such approach causes some overstatement of actual pharmacotherapy costs incurred for the treatment of cardiovascular diseases due to the fact that some of drugs from ATC class C are used for the treatment of arterial hypertension or essential hypertension. However, based on publicly available data, it is not possible to determine the percentage of patients using drugs in the above-mentioned indications. Data on the costs of non-reimbursement medications are not publicly available, and therefore not included in the analysis.

#### Medical Services

The costs of medical services incurred in the treatment of cardiovascular diseases were estimated on the basis of the value of NHF contracts concluded by individual NHF’ Provincial Departments in a given year (the data are divided into the service provider, type of service and the name of the contracted product). The following types of medical services were included in the analysis:

- outpatient specialist care,
- hospital treatment,
- medical rehabilitation,
- health services contracted separately.

The organization of services assumes that every procedure financed from public funds is reported by the healthcare provider to the appropriate NHF’ Provincial Department, where after checking the correctness of the information provided, appropriate financial resources are paid (Rutkowski et al., 2015). Some healthcare services are financed in the form of a lump sum (the service provider receives funds from the NHF before and not after the patient’s treatment), and their value is not included in the contracts reported by the public payer. Thus, total costs of hospital treatment estimated in this publication may be slightly underestimated.

### Indirect Costs

Indirect costs are divided into four categories. The first (absenteeism) concerns expenses related to the employee’s absence from work due to CVD. The second (presenteeism) takes into account the cost of loss in productivity of employee, who despite CVD, remains present at work, but whose performance due to illness is reduced. The third category of costs (incapacity for work) relates to expenses incurred as a result of the patient’s temporary or permanent inability to work. The last, fourth category is associated with the premature death of the patient due to CVD.

#### General Assumptions

We estimated the indirect costs of CVD using the human capital method, modified in accordance with the recommendations presented in the document “Methodology for Measuring Indirect Cost of Illness in the Polish Healthcare System published in 2013” (EY, 2013).

The unit of lost productivity is expressed in the Gross Domestic Product (GDP) per working person, however, the difference between the concept of an employee and a working person should be taken into account (as the former does not include self-employment). In addition to lost labor productivity, this measure also expresses the lost productivity of a summarized production input, which with high probability could be more efficiently utilized by a healthy individual.

In connection with the occurrence of the phenomenon of diminishing marginal labor productivity, it was necessary to adopt a correction factor in the analysis. We used the value of 0.65 in accordance with the position adopted by the European Commission for EU-15 countries over the period of 1960 to 2003 (Leal et al., 2006), which corresponds to the marginal relation to the average labor efficiency. Thus, the productivity unit was calculated according to the formula:

(1) $$Unit\;of\;Productivity=GDP_{w\;}\cdot 0.65.$$

We also implied the economic and demographic indicators from the years 2015 to 2017. (see **Appendix 1**). Each of the indirect costs borne in the future are expressed in terms of 2017 GDP.

#### Absenteeism

The costs associated with an unplanned absence from work due to the cardiovascular diseases were estimated taking into account data from the SII Statistical Portal in Poland. We used the number of days of sick leave reported for ICD-10: I00-I99 codes from 2015 to 2017 (data on informal leave days were not included in the analysis). In total CVD accounted for 12,233,203, 12,239,697, and 12,210,793 sickness leave days in 2015, 2016, and 2017, consecutively (see **Appendix 2**). Assuming that the year includes 252 business days, we calculated a comparable number of working person-years lost as CVD-related absenteeism (*A* ^(^ *^y^* ^)^). In the next step, we determined the indirect cost of absenteeism (*IC^A^*) using the data from **Appendix 1** on the GDP per working person according to the equation:

(2) $$IC^{A}=GDP_{w\;}\cdot 0.65\cdot A^{\left(y\right)}.$$

#### Presenteeism

CVD have a significant impact on the quality of life and work of the patient. Given the subjective nature of presenteeism, estimation of the single patient (at working-age) unit impact on the productivity is difficult. In our analysis we adopted the assumption presented in the Kotseva 2018^2^, whose authors assumed a loss of 10 working days a year for a representative patient in comparison to a healthy person. It should be borne in mind that this assumption may be subject to some error, as the questionnaire was not carried out in the Polish population.

We combined the above impact on productivity with data on the population of patients with cardiovascular diseases in Poland, derived from the POLKARD report, according to which, the total number of CVD patients in Poland amounts to 1,160,667 (this value also includes unregistered and untreated CVD patients)^3^. We then narrowed this value to the working-age population with CVD by taking into account the percentage of working persons (own estimate, see **Appendix 4**). Subsequently, we estimated the total number of person-years lost per year due to presenteeism (*P* ^(^ *^y^* ^)^) by multiplying the number of working people with CVD (*N*) and the impact on the productivity in relation to the number of business days per year:

(3) $$P^{\left(y\right)}\;=\;\frac{N\cdot 10}{252},$$

Finally, the total indirect cost of presenteeism (*IC^P^*) was calculated as:

(4) $$IC^{P}=GDP_{w\;}\cdot 0.65\cdot P^{\left(y\right)}.$$

#### Incapacity for Work

Total costs related to incapacity for work (pension) were determined using data from SII regarding the number of people diagnosed with ICD-10: I00-I99 and taking into account following categories: i) incapacity for independent existence, ii) total incapacity for work, iii) partial incapacity for work (see **Appendix 5**). Total indirect cost of incapacity for work (*IC^I^*) was estimated as the sum of the cost of permanent (*IC^I,P^*) and temporary incapacity (*IC^I,T^*):

(5) $$IC^{I}=IC^{I,P}+IC^{I,T}.$$

##### Permanent Incapacity

The analysis assumes that permanent incapacity for work includes people who are completely unable to work or who require additional external care for existence. In order to calculate indirect costs for this cost category, we have estimated the following features:

1. the number of years the patient would have theoretically worked from the time of decision of incapacity for work to retirement age if he/she was healthy (based on the patient’s age and retirement age in Poland);
2. the probability of survival in the above period of time (based on the patient’s age and life expectancy tables according to the CSO);
3. the probability that a patient would have worked provided that he/she had not suffered from a CVD or deceased for any reason.

It has been presumed that working age begins at the age of 19, thanks to which compliance with available statistics was possible. The expected number of further potential working years *E*(X_n_) results from the statutory retirement age *k(n)*, and the age at which the decision on incapacity for work was made *n*:

(6) $$E\left({\text{X}}_{\text{n}}\right)\;=\Sigma_{i=1}^{k\left(n\right)-n-1}i\cdot p_{\left(n+i\right)}+[k(n)-n](1-\Sigma_{i=1}^{k-n}p_{n+i}),$$

where:

*p_n_*_+1_ – probability of death at the age of *n+i* (see **Appendix 6**).

The calculations take into account pension reforms in Poland that took place in the analyzed period (the age of retirement was subject to change), which may have a slight impact on the indirect costs of permanent incapacity for work or mortality.

As data on distribution of CVD-related decisions over *n* is not available, we assumed that the distribution of all decisions (see **Appendix 7**) is sufficiently representative and included it in the calculations of the average expected value of lost working years.

We calculated the average number of lost working years per patient subject to decision ($\bar{x}$) taking into account expected number of further potential working years and *α_n_* — a share of the cohort aged *n* (in the patient population subject to decisions, Σ_n_ α_n_ = 1). Age distribution was used in order to calculated statistical number of potential working years (up to 65).

(7) $$\bar{X}=\Sigma_{n=19}^{65}\;E(X_{n})\cdot \alpha_{n},$$

In the next step, the number of lost working years due to permanent incapacity for work resulting form CVD (*IP* ^(^ *^y^* ^)^) was estimated as:

(8) $$\;IP^{\left(y\right)}=N^{I,P}\cdot \bar{X}\cdot \gamma ,$$

where:

N_n,t_ — annual number of permanent inability decisions attributed to CVD,

*γ* — fraction of working population in total population aged 19–65 (see **Appendix 7**).

Finally, the total loss of production due to permanent incapacity to work was calculated according to the formula as in (2) and (4):

(9) $$IC^{I,P}=GDP_{w\;}\cdot 0.65\cdot IP^{\left(y\right)}.$$

##### Temporary Incapacity

In order to estimate the costs of temporary incapacity for work, SII data on ICD-10 codes: I00-I99 were used, according to which the average time of temporary inability to work amounts to 16 months (1.33 years). Partial incapacity for work is parallel to 75% loss of working potential. This is in line with the social security statute that sets the benefit for partial disability at 75% of the benefit associated with full disability. The number of working person-years due to temporary incapacity for work, (*IT* ^(^ *^y^* ^)^), is calculated taking into account the annual number of temporary disability decisions attributed to CVD (*N* ^(^ *^I,T^* ^)^):

(10) $$IT^{\left(y\right)}=N^{I,T}\cdot 1.33\cdot 0.75,$$

Similarly to (9), the indirect costs component related to temporary incapacity for work is calculated as:

(11) $$IC^{I,T}=GDP_{w\;}\cdot 0.65\cdot IT^{\left(y\right)}.$$

#### Mortality

The indirect cost related to mortality was estimated as the sum of lost potential years of work taking into account the moment of premature death due to CVD and the expected moment of retirement. We also took into account the low probability of dying from causes other than CVD during this period. CSO data from 2015 to 2017 referring to the number of CVD-related deaths (determined on the basis of ICD-10 codes, see **Appendix 9**) were used, which were then transformed into the number of lost years of work lost for a given person with CVD and his/her age death (*n*) according to the formula (11) —see **Appendix 8**. Finally, the total number of lost working years (*M* ^(^ *^y^* ^)^) was calculated as a sum over all the cohorts with *N^M,n^* being the number of people that died at the age of *n* due to CVD:

(12) $$M^{\left(y\right)}\;=\;\Sigma_{n=19}^{65}N^{M,n}\cdot E(X_{n}),$$

Finally, the indirect cost related to mortality was defined as:

(13) $$IC^{M}=GDP_{w\;}\cdot 0.65\cdot M^{\left(y\right)}._{\;}$$

Since data on mortality in individual age groups in 2017 were not available, we assumed their invariance for previous years for the purposes of this calculation.

## Results

### Direct Costs

The total estimated direct cost of cardiovascular diseases (including drugs reimbursement and medical services costs) in Poland amounted to 1.5 bn EUR in 2015, 1.5 bn EUR in 2016 and 1.3 bn EUR in 2017. The main factors determining costs are hospital treatment and drugs (**Table 2**).

**Table 2:** Direct cost of cardiovascular diseases (I00-I99) in Poland estimates for 2015–2017 years (%, PLN, EUR).

Cost category	Cardiovascular diseases											
	2015			2016			2017			2015–2017 (average)		
	%	Direct cost (PLN)	Direct cost (EUR)	%	Direct cost (PLN)	Direct cost (EUR)	%	Direct cost (PLN)	Direct cost (EUR)	%	Direct cost (PLN)	Direct cost (EUR)
Drugs	41%	2,607,604,086	610,966,281	41%	2,570,295,952	602,224,918	47%	2,689,567,724	630,170,507	43%	2,622,489,254	614,453,902
Hospital treatment	51%	3,278,249,297	768,099,648	51%	3,226,052,337	755,869,807	45%	2,552,684,406	598,098,502	49%	3,018,995,347	707,355,986
Outpatient specialist care	6%	352,217,904	82,525,282	6%	359,403,818	84,208,954	6%	335,257,344	78,551,393	6%	348,959,689	81,761,876
Medical rehabilitation	2%	133,325,312	31,238,358	2%	137,904,612	32,311,296	2%	130,037,131	30,467,931	2%	133,755,685	31,339,195
Health services contracted separately	0%	144	34	0%	60,926	14,275	0%	30,090	7,050	0%	30,386	7,120
Total	100%	**6,371,396,743**	**1,492,829,602**	100%	**6,293,717644**	**1,474,629,251**	100%	**5,707,576,694**	**1,337,295,383**	100%	**6,124,230,360**	**1,434,918,079**

Source: Own elaboration based on NHF data.

Drugs cost (both reimbursement and patient co-payment) increased from approximately 611.0 mln EUR to 630.2 mln EUR per year. In case of medical services costs, we observe a decrease in their value in 2017 compared to 2015. The decrease in 2017 compared to the previous year is greater than the corresponding decrease in 2016—this is most likely due to regulatory changes that occurred in the way of contracting certain health services in 2017 that could have significantly underestimated the actual cost. It is worth noting that out of all direct medical services costs, the largest share is the cost of hospital treatment (86% on average).

### Indirect Costs

The estimated total indirect costs of cardiovascular diseases in Poland from 2015 to 2017 were respectively 8.0 bn EUR, 7.1 bn EUR and 6.8 bn EUR. These costs correspond to the loss of 494.4 thousand person-years in 2015, 432.5 thousand person-years in 2016 and 406.7 thousand person-years in 2017. Of the four categories included, costs related to incapacity for work and mortality have the largest share (**Tables 3** and **4**).

**Table 3:** Indirect cost of absenteeism, presenteeism, permanent and temporary incapacity for work and mortality caused by cardiovascular diseases in Poland estimates for the 2015–2017 years (%, PLN, EUR).

Cost category	Cardiovascular diseases												
	2015			2016			2017			2015–2017 (average)			
	%	Indirect cost (PLN)	Indirect cost (EUR)	%	Indirect cost (PLN)	Indirect cost (EUR)	%	Indirect cost (PLN)	Indirect cost (EUR)	%	Indirect cost (PLN)	Indirect cost (EUR)
Absenteeism	11%	3,830,409,595	897,471,789	13%	3,841,977,207	900,182,101	14%	3,986,889,601	934,135,333	12%	3,886,425,468	910,596,408
Presenteeism	6%	1,963,017,888	459,938,587	7%	2,029,629,244	475,545,746	7%	2,168,768,754	508,146,381	7%	2,053,805,295	481,210,238
Permanent and temporary incapacity for work (pension)	48%	16,621,792,347	3,894,515,545	47%	14,215,992,708	3,330,832,406	49%	14,320,643,443	3,355,352,259	48%	15,052,809,499	3,526,900,070
Mortality	35%	12,074,517,576	2,829,080,969	34%	10,225,829,242	2,395,930,001	30%	8,755,975,186	2,051,540,578	33%	10,352,107,335	2,425,517,182
Total	100%	**34,489,737,406**	**8,081,006,890**	100%	**30,313,428,401**	**7,102,490,253**	100%	**29,232,276,983**	**6,849,174,551**	100%	**31,345,147,597**	**7,344,223,898**

Source: Own elaboration based on CSO and SII data.

**Table 4:** The number of working years lost to cardiovascular diseases in Poland—estimates for the 2015–2017 years.

Cost category	Cardiovascular diseases		
	2015	2016	2017
	The number of lost working years	The number of lost working years	The number of lost working years
Absenteeism	48,544	48,570	48,456
Presenteeism	24,878	25,659	26,359
Permanent and temporary incapacity for work	202,017	172,777	174,049
Mortality	218,989	185,460	158,802
TOTAL	494,428	432,466	407,665

Source: Own elaboration based on CSO and SII data.

Among the four analyzed categories of indirect costs related to CVD, the largest share (almost half of the total expenses) constitute costs related to the patient’s incapacity for work – estimates show a decrease from EUR 3.9 bn in 2015 to EUR 3.5 bn in 2017. Indirect costs of CVD-related deaths are the second largest category of costs and also have a downward trend (from 2.8 EUR bn in 2015 to EUR 2.1 bn in 2017). In the case of the remaining categories of costs (absenteeism and presenteeism), the reverse trend is noticeable, i.e. an increase in expenses incurred in subsequent years of analysis. According to our estimates, the cost of absence from work of a patient with CVD in Poland was 897.5 mln EUR in 2015 and 934.1 mln EUR in 2017. Similarly, the costs associated with reduced patient performance at work due to CVD increased from 459.9 mln EUR in 2015 to 508.1 mln EUR in 2017.

### Total Cost

The total annual average costs of CVD in Poland (both direct and indirect) amount to 37.5 bn PLN, (8.8 bn EUR), and it is 1.89% of GDP. The total estimated indirect costs of CVD amount to 31.3 bn PLN (7.3 bn EUR) and significantly exceeds the direct medical costs, which amount to 6.1 bn PLN (1.4 bn EUR) (**Table 5**).

**Table 5:** Direct and indirect cost of cardiovascular diseases in Poland estimates for the 2015–2017 years (%, PLN, EUR).

Cost category	Cardiovascular diseases											
	2015			2016			2017			2015–2017 (average)		
	%	(PLN)	(EUR)	%	(PLN)	(EUR)	%	(PLN)	(EUR)	%	(PLN)	(EUR)
**Direct cost**	16%	6,371,396,743	1,492,829,602	17%	6,293,717,644	1,474,629,251	16%	5,707,576,694	1,337,295,383	16%	6,124,230,360	1,434,918,079
**Indirect cost**	84%	34,489,737,406	8,081,006,890	83%	30,313,428,401	7,102,490,253	84%	29,232,276,983	6,849,174,551	84%	31,345,147,597	7,344,223,898
**Total**	100%	**40,861,134,148**	**9,573,836,492**	100%	**36,607,146,045**	**8,577,119,505**	100%	**34,939,853,677**	**8,186,469,934**	100%	**37,469,377,957**	**8,779,141,977**

Source: Own elaboration based on NHF, CSO and SII data.

It should be noticed that the direct as well as indirect cost appears to be gradually decreasing in time. Direct costs went from 6.4 bn PLN (1.5 bn EUR) in 2015 to 5.7 bn PLN (1.3 bn EUR) in 2017 and indirect costs from 34.5 bn PLN (8.1 bn EUR) in 2015 to 29.2 bn PLN (6.8 bn EUR) in 2017 (**Table 5**).

## Discussion

Treatment of cardiovascular diseases is one of the biggest challenges for health care systems. According to various data, they cost the European economy 160 bn to 210 bn EUR a year (Paczkowska et al., 2014). Due to the importance of this problem, there are a number of papers discussing the cost of CVD in different European countries available (Dziki et al., 2017). These studies used both a top-down and a bottom-up approach to assess the resources used. However, it is not possible to compare the financial expenditure for CVD treatment in individual countries due to the different data collection methodologies, the variety of data sources used and the inclusion of different cost categories.

Our analysis focused on direct and indirect costs of CVD from 2015 to 2017 from the payer and societal perspective in Poland. We used data from Polish institutions (NHF, CSO, SII) and the methodology presented in the EY (Ernst & Young Global Limited Liability Partnership, commonly known as Ernst & Young) report on the methodology for measuring indirect costs in the Polish healthcare system (EY, 2013). To our knowledge, in terms of the applied methodology, this is the first study to estimate both direct and indirect costs associated with CVD in Poland. According to the results obtained, the total costs of CVD from 2015 to 2017 fluctuated between 34.9 bn PLN (8.2 bn EUR) and 40.9 bn PLN (9.6 bn EUR). The increase in total costs associated with CVD in Poland correlates with a visible trend in the field of epidemiology, the current data indicate an increase in the number of patients in subsequent years (Gańczak et al., 2020). Indirect costs constituted 84% of the total costs (approximately 31.3 bn PLN, 7.3 bn EUR) and significantly exceeded direct costs in the same period (approximately 6.1 bn PLN, 1.4 bn EUR). This task is primarily with the specificity of the diseases included in the analysis. Most of the CVD diseases are chronic and require long-term therapy, which is not conducted in a hospital or outpatient setting and thus does not generate direct costs in a continuous manner (hospitalizations for CVD causes are mainly associated with exacerbation of the disease and after a few days, usually ill returns home). The significant part of the analyzed disease entities is associated with inability to work (permanent or temporary) and thus reduced productivity. Also, the high mortality rate due to CVD in Poland means that indirect costs have the largest part in the total costs related to the disease.

However, it seems that the total costs related to CVD in Poland may be higher than estimated, because our calculations do not take into account certain categories of costs (e.g. data on primary health care, transport costs in case of emergency, costs of informal care) due to the lack of access to appropriate (reliable) data. Another limitation of the analysis is also the lack of data from the private sector (e.g. paid medical visits, medicines without reimbursement) as such data are not available in the public domain.

The higher total direct cost incurred by the National Health Fund for medical services in the field of cardiology (ICD-10 codes: from I00 to I99) is indicated by the publication, in which authors used the NHF internal databases and analyzed the size and structure of services (related to cardiovascular diseases) financed from public funds in Poland (Rutkowski et al., 2015). Results indicate an increase in the total cost of services from 6.5 bn PLN in 2009 to 7.8 bn PLN in 2014. At the same time, the hospital treatment cost increased from 2.53 bn PLN to 2.99 bn PLN. While costs of hospital treatment in 2012–2014 from Rutkowski 2015 are at a similar level to those estimated in our analysis in 2015–2017, the total cost of medical services is higher. The results show that in years 2009–2014 the value of contracts in hospital treatment constituted from 38.3 to 40.4% of the total NHF expenditure incurred on cardiology. Assuming that the relation between the amount of contracts for hospital treatment and total NHF expenditure in years 2015–2017 was at a similar level to that in 2012–2014 (38.9% on average), the total estimated cost of cardiovascular diseases (incl. drugs) in Poland would amount to 11.0 bn PLN in 2015, 10.87 bn PLN in 2016 and 9.25 bn PLN in 2017—nearly 1.7 times higher than those estimated in our analysis.

According to the 10-year resource utilization and costs in the US it is suggested that CVD-related economic burdens on the health care system will increase, taking into consideration cumulative medications, office visits, diagnostic procedures, coronary revascularization, and hospitalizations (Calafiero and Jané-Llopis, 2011; Heidenreich et al., 2011; World Health Organization. (WHO), 2014; Siqueira et al., 2017; Williams et al., 2018). In this case, the 10-year longitudinal patterns of CVD cost after the screening of more than 6.8 thousand asymptomatic participants from the MESA (Multi-Ethnic Study of Atherosclerosis) registry accounted for 155 mln USD. The data comes from AHA reports and clearly indicate the productivity losses and medical costs of CVD are projected to increase, taking into account the US population, 555 bn USD in 2015 to 1.1 trillion USD in 2035 (Siqueira et al., 2017). Therefore, the corresponding costs of informal caregiving for patients suffering from CVD diseases were calculated at 61 bn USD in 2015 with appropriate projection to increase to 128 bn USD in 2035 (Calafiero and Jané-Llopis, 2011). Concurrently, the informal caregiving costs of patients with stroke establish more than 50% of the whole costs of CVD informal caregiving, which in 2015 amounted to over 30 bn USD and is expected to amount to more than twice as much in 2035. Similar findings, regarding proportions of cost component of CVD, were observed in the UK where health care costs accounted for 60%, followed by productivity losses accounting for 23%, with 17% due to informal care related costs, which accounted £29.1 bn in 2004 (Williams et al., 2018). According to the available data from the EU the CVD costs were estimated to be 169 bn Euro annually, including 62% healthcare costs, 21% productivity losses costs, and 17% informal care costs (Heidenreich et al., 2011).

As mentioned earlier, due to differences in methods and data used in individual studies described above, cost comparison between countries is limited. Nevertheless, the direct and indirect costs associated with CVD in Poland estimated in this publication may be used in the future, e.g. while conducting health technology assessment (for drugs or health/medical products) and, as a result, contribute to the improvement of both the state of health of the Polish society and the quality of life patients with CVD.

## Conclusion

1. CVD treatment is one of the biggest challenges for the healthcare system in Europe, with the estimation of total costs being difficult (due to the limited availability of data) as well as comparison between individual countries (due to the variety of methodology used).
2. Estimated direct costs of CVD in Poland in years 2015–2017 amounted to 1.3–1.5 bn EUR and were mainly related to expenses incurred for hospital treatment and drug reimbursement.
3. Permanent and temporary incapacity for work (pension) is the largest cost generator of the CVD indirect costs in Poland, which in total were over 5 times higher than direct costs estimated in the same period.

## Data Availability Statement

The raw data supporting the conclusions of this article will be made available by the authors, without undue reservation, to any qualified researcher.

## Author Contributions

AM—conception and design, analysis and interpretation of the data, drafting of the paper and revising it critically for intellectual content, final approval of the version to be published. ER—drafting of the paper and revising it critically for intellectual content. ŁAP—interpretation of the data, revising paper critically for intellectual content. JJ—drafting of the paper and revising it critically for intellectual content. MF-N—drafting of the paper and revising it critically for intellectual content. MS-G—drafting of the paper and revising it critically for intellectual content. DO—drafting of the paper and revising it critically for intellectual content. MN—drafting of the paper and revising it critically for intellectual content. AS—revising paper critically for intellectual content, final approval of the version to be published. All authors agree to be accountable for all aspects of the work.

## Conflict of Interest

The authors declare that the research was conducted in the absence of any commercial or financial relationships that could be construed as a potential conflict of interest. 

## Footnotes

1 The weighted average exchanges rates: 1 EUR: 2015 (4.18), 2016 (4.36), 2017 (4.26); average exchange rate in 2015–2017 years (1 EUR – 4.27 PLN). Available from: http://www.nbp.pl/home.aspx?f=/kursy/arch_a.html

2 K Kotseva, L Gerlier, E Sidelnikov, L Kutikova, M Lamotte, L Annemans, P Amarenco. Patient and caregiver productivity loss and indirect costs associated with cardiovascular events in Europe. European Heart Journal, Volume 39, Issue suppl_1, August 2018, ehy565.P2561, https://doi.org/10.1093/eurheartj/ehy565.P2561. https://academic.oup.com/eurheartj/article/39/suppl_1/ehy565.P2561/5081485/

3 “Program Profilaktyki i Leczenia Chorób Układu Sercowo-Naczyniowego POLKARD na lata 2017–2020”. https://www.gov.pl/documents/292343/436711/polkard-2017-2020-aktualizacja+pdf.pdf/f9a56b63-9ad2-060d-40f1-f84ea520f3b8?fbclid=IwAR0iWqmtJJ:AlalaT87pvHIcGW-JvNIsI_LKWyE8LzScvPBntCV2i_7bDY

## References

[B1] Boles M. Pelletier B. Lynch W. (2004). The relationship between health risks and work productivity. J. Occup. Environ. Med. 46 (7), 737–745. 10.1097/01.jom.0000131830.45744.97 15247814

[B2] Calafiero E. T. Jané-Llopis E. (2011). The Global Economic Burden of Noncommunicable diseases (London: World Ecomnomic Forum), 1–48.

[B3] Czech M. Opolski G. Zdrojewski T. Dubiel J. S. Wizner B. Bolisęga D. (2013). The costs of heart failure in Poland from the public payer’s perspective. Polish programme assessing diagnostic procedures, treatment and costs in patients with heart failure in randomly selected outpatient clinics and hospitals at different levels of care: POLKARD. Kardiol. Pol. 71 (3), 224–232. 10.5603/KP.2013.0032 23575775

[B4] Dziki B. Miechowicz I. Iwachów P. Kuzemczak M. Kałmucki P. Szyszka A. (2017). Cath lab costs in patients undergoing percutaneous coronary angioplasty - detailed analysis of consecutive procedures. Kardiol. Pol. 75 (9), 914–921. 10.5603/KP.a2017.0098 28541592

[B5] EY (2013). Metodyka pomiaru kosztów pośrednich w polskim systemie ochrony zdrowia (Warszawa: SPRAWNE PAŃSTWO EY).

[B6] Gańczak M. Miazgowski T. Kożybska M. Kotwas A. Korzeń M. Rudnicki B. (2020). Changes in disease burden in Poland between 1990-2017 in comparison with other Central European countries: A systematic analysis for the Global Burden of Disease Study 2017. PloS One 15 (3), e0226766. 10.1371/journal.pone.0226766 32119685 PMC7051048

[B7] Gaziano T. A. Bitton A. Anand S. Abrahams-Gessel S. Murphy A. (2010). Growing epidemic of coronary heart disease in low- and middle-income countries. Curr. Probl. Cardiol. 35 (2), 72–115. 10.1016/j.cpcardiol.2009.10.002 20109979 PMC2864143

[B8] Graham I. Atar D. Borch-Johnsen K. Boysen G. Burell G. Cifkova R. (2007). European guidelines on cardiovascular disease prevention in clinical practice: executive summary: Fourth Joint Task Force of the European Society of Cardiology and Other Societies on Cardiovascular Disease Prevention in Clinical Practice (Constituted by representatives of nine societies and by invited experts). Eur. Heart J. 28 (19), 2375–2414. 10.1093/eurheartj/ehm316 17726041

[B9] Heidenreich P. A. Trogdon J. G. Khavjou O. A. Butler J. Dracup K. Ezekowitz M. D. (2011). American Heart Association Advocacy Coordinating Committee; Stroke Council; Council on Cardiovascular Radiology and Intervention; Council on Clinical Cardiology; Council on Epidemiology and Prevention; Council on Arteriosclerosis; Thrombosis and Vascular Biology; Council on Cardiopulmonary; Critical Care; Perioperative and Resuscitation; Council on Cardiovascular Nursing; Council on the Kidney in Cardiovascular Disease; Council on Cardiovascular Surgery and Anesthesia, and Interdisciplinary Council on Quality of Care and Outcomes Research. Forecasting the future of cardiovascular disease in the United States: a policy statement from the American Heart Association. Circulation 123 (8), 933–944. 10.1161/CIR.0b013e31820a55f5 21262990

[B10] Islami F. Mańczuk M. Vedanthan R. Vatten L. Polewczyk A. Fuster V. (2011). A cross-sectional study of cardiovascular disease and associated factors. Ann. Agric. Environ. Med. 18 (2), 255–259. 22216792

[B11] Kuehn B. M. (2013). Costs of cardiac care likely to increase, despite advances in prevention, care. JAMA 310 (19), 2029. 10.1001/jama.2013.282805 24240914

[B12] Leal J. Luengo-Fernández R. Gray A. Petersen S. Rayner M. (2006). Economic burden of cardiovascular diseases in the enlarged European Union. Eur. Heart J. 27 (13), 1610–1619. 10.1093/eurheartj/ehi733 16495286

[B13] Ludność Stan i struktura oraz ruch naturalny w przekroju terytorialnym w 2018 r. Stan w dniu 30 VI Population. Size and structure and vital statistics in Poland by territorial division in 2018. As of June, 30.https://stat.gov.pl/obszary-tematyczne/ludnosc/ludnosc/ludnosc-stan-i-struktura-oraz-ruch-naturalny-w-przekroju-terytorialnym-w-2018-r-stan-w-dniu-30-vi,6,24.html.

[B14] Mensah G. A. Brown D. W. (2007). An overview of cardiovascular disease burden in the United States. Health Aff. (Millwood) 26 (1), 38–48. 10.1377/hlthaff.26.1.38 17211012

[B15] Milani R. V. Lavie C. J. (2009). Impact of worksite wellness intervention on cardiac risk factors and one-year health care costs. Am. J. Cardiol. 104 (10), 1389–1392. 10.1016/j.amjcard.2009.07.007 19892055

[B16] OECD/European Observatory on Health Systems and Policies (2017). “Poland: Country Health Profile 2017”, in State of Health in the EU. (Brussels: OECD Publishing, Paris/European Observatory on Health Systems and Policies). 10.1787/9789264283510-en

[B17] Paczkowska A. Koligat D. Nowakowska E. Hoffmann K. Bryl W. (2014). Analysis of direct costs of hypertension treatment among adolescents in Poland. Acta Pol. Pharm. 71 (1), 197–203. 24779208

[B18] Poloński L. Gasior M. Gierlotka M. Kalarus Z. Cieśliński A. Dubiel J. S. (2007). Polish Registry of Acute Coronary Syndromes (PL-ACS). Characteristics, treatments and outcomes of patients with acute coronary syndromes in Poland. Kardiol. Pol. 65 (8), 861–872. discussion 873-4. 17853315

[B19] Rutkowski D. Śliwczyński A. Brzozowska M. Czeleko T. Jędrzejczyk T. (2015). Wielkość i struktura świadczeń związanych z chorobami układu krążenia finansowanych ze środków publicznych w Polsce. https://bip.stat.gov.pl/files/gfx/bip/pl/zamowieniapubliczne/426/248/1/81_gp_rrl_2015_monografia_kardiologiczna.pdf.

[B20] Siqueira A. S. E. Siqueira-Filho A. G. Land M. G. P. (2017). Analysis of the Economic Impact of Cardiovascular Diseases in the Last Five Years in Brazil. Arq. Bras. Cardiol. 109 (1), 39–46. 10.5935/abc.20170068 28591251 PMC5524474

[B21] Song X. Quek R. G. Gandra S. R. Cappell K. A. Fowler R. Cong Z. (2015). Productivity loss and indirect costs associated with cardiovascular events and related clinical procedures. BMC Health Serv. Res. 15, 245. 10.1186/s12913-015-0925-x 26104784 PMC4478719

[B22] Sović N. Pająk A. Jankowski P. Duenas A. Kawecka-Jaszcz K. Wolfshaut-Wolak R. (2013). Cost-effectiveness of a cardiovascular disease primary prevention programme in a primary health care setting. Results of the Polish part of the EUROACTION project. Kardiol. Pol. 71 (7), 702–711. 10.5603/KP.2013.0157 23907903

[B23] Suwalski P. Kowalewski M. Jasiński M. Staromłyński J. Zembala M. Widenka K. (2018). KROK Investigators. Survival after surgical ablation for atrial fibrillation in mitral valve surgery: Analysis from the Polish National Registry of Cardiac Surgery Procedures (KROK). J. Thorac. Cardiovasc. Surg. 10.1016/j.jtcvs.2018.07.099. pii: S0022-5223(18)32321-3. 30314688

[B24] Tarride J. E. Lim M. DesMeules M. Luo W. Burke N. O’Reilly D. (2009). A review of the cost of cardiovascular disease. Can. J. Cardiol. 25 (6), e195–e202. 10.1016/S0828-282X(09)70098-4 19536390 PMC2722492

[B25] Williams J. Allen L. Wickramasinghe K. Mikkelsen B. Roberts N. Townsend N. (2018). A systematic review of associations between non-communicable diseases and socioeconomic status within low- and lower-middle-income countries. J. Glob. Health 8 (2):20409. 10.7189/jogh.08.020409 PMC6076564 30140435

[B26] World Economic Forum (2011). The Global Economic Burden of Non-communicable Diseases (Harvard School of Public Health).

[B27] World Health Organization. (WHO) (2014). Global status report on noncommunicable disease 2014 (Geneva: World Health Organization).

