# Supplementary material for: Economic Costs of Cardiovascular Diseases in Poland Estimates for 2015–2017 Years
Source: Front Pharmacol. 2020 Sep 8;11:1231. doi: 10.3389/fphar.2020.01231 (PMC7506040; doi:10.3389/fphar.2020.01231)
Supplement: Supplementary file 1 [file DataSheet_1.docx]

# **SUPPLEMENTARY DATA**

# **ABSENTEEISM**

Appendix 1. Economic and demographic indicators as of 2015-2017 years for Poland.

| Indicator | 2015 | 2016 | 2017 |
| --- | --- | --- | --- |
| 1. GDP at current market prices* | 421,797 EUR  469,997 USD | 436,062 EUR  485,892USD | 465,963 EUR  519,210 USD |
| 1. Working population, end of period** | 14,829,800 | 15,293,300 | 15,710,800 |
| 1. GDP per 1 working person, (1)/(2), $\boldsymbol{GDP}_{\boldsymbol{w}}$*** | 28,443 EUR  31,693 USD | 28,513 EUR  31,772 USD | 29,659 EUR  33,048 USD |
| 1. Total population in Poland† | 38,437,200 | 38,433,000 | 38,433,600 |
| 1. Fraction of working population in Poland (2) / (4) † | 0.39 | 0.40 | 0.41 |
| 1. Fraction of working population in the population aged 19-65 ($\boldsymbol{\gamma}$)* | 0.62 | 0.64 | 0.67 |

*CSO (macroeconomic indicators/National Accounts ESA 2010).

**CSO (macroeconomic indicators/labour market).

*** Own elaboration.

†CSO (population/life expectancy).

Appendix 2. Sickness absenteeism caused by cardiovascular diseases (ICD-10: I00-I99) in the 2015-2017 years in Poland.

| The code according to ICD-10 | | 2015 | 2016 | 2017 |
| --- | --- | --- | --- | --- |
|  |  | Sickness leave days | Sickness leave days | Sickness leave days |
|  |  |  |  |  |
|  |  |  |  |  |
| I00 | Rheumatic fever without mention of heart involvement | 39 610 | 39 878 | 38 398 |
| I01 | Rheumatic fever with heart involvement | 24 599 | 25 372 | 24 792 |
| I02 | Rheumatic chorea | 29 679 | 27 902 | 27 275 |
| I05 | Rheumatic mitral valve diseases | 11 523 | 11 867 | 8 111 |
| I06 | Rheumatic aortic valve diseases | 89 888 | 91 154 | 88 501 |
| I07 | Rheumatic tricuspid valve diseases | 2 834 | 2 211 | 2 587 |
| I08 | Multiple valve diseases | 12 442 | 10 415 | 9 896 |
| I09 | Other rheumatic heart diseases | 1 787 | 1 573 | 1 450 |
| I10 | Essential (primary) hypertension | 2 723 698 | 2 520 548 | 2 404 838 |
| I11 | Hypertensive heart disease | 388 246 | 360 856 | 334 778 |
| I12 | Hypertensive renal disease | 8 933 | 9 477 | 8 424 |
| I13 | Hypertensive heart and renal disease | 4 184 | 3 389 | 3 220 |
| I15 | Secondary hypertension | 15 970 | 16 372 | 15 764 |
| I20 | Angina pectoris | 484 209 | 491 366 | 463 246 |
| I21 | Acute myocardial infarction | 457 934 | 488 504 | 538 344 |
| I22 | Subsequent myocardial infarction | 8 946 | 8 461 | 9 899 |
| I23 | Certain current complications following acute myocardial infarction | 5 273 | 4 234 | 4 683 |
| I24 | Other acute ischaemic heart diseases | 17 325 | 14 689 | 14 483 |
| I25 | Chronic ischaemic heart disease | 1 959 752 | 1 893 126 | 1 905 295 |
| I26 | Pulmonary embolism | 103 981 | 109 009 | 118 572 |
| I27 | Other pulmonary heart diseases | 9 336 | 11 391 | 12 479 |
| I28 | Other diseases of pulmonary vessels | 3 398 | 3 023 | 2 906 |
| I30 | Acute pericarditis | 17 106 | 23 056 | 25 066 |
| I31 | Other diseases of pericardium | 8 543 | 8 638 | 8 945 |
| I32 | Pericarditis in diseases classified elsewhere | 18 327 | 21 590 | 22 076 |
| I33 | Acute and subacute endocarditis | 14 134 | 13 891 | 12 466 |
| I34 | Nonrheumatic mitral valve disorders | 63 000 | 65 652 | 70 095 |
| I35 | Nonrheumatic aortic valve disorders | 81 994 | 90 502 | 97 586 |
| I36 | Nonrheumatic tricuspid valve disorders | 3 896 | 4 549 | 3 989 |
| I37 | Pulmonary valve disorders | 7 641 | 6 842 | 7 135 |
| I38 | Endocarditis, valve unspecified | 5 392 | 7 556 | 6 691 |
| I39 | Endocarditis and heart valve disorders in diseases classified elsewhere | 6 965 | 7 268 | 6 754 |
| I40 | Acute myocarditis | 48 331 | 53 175 | 54 216 |
| I41 | Myocarditis in diseases classified elsewhere | 8 340 | 7 944 | 8 450 |
| I42 | Cardiomyopathy | 119 836 | 129 065 | 106 482 |
| I43 | Cardiomyopathy in diseases classified elsewhere | 4 475 | 4 544 | 4 626 |
| I44 | Atrioventricular and left bundle-branch block | 40 207 | 42 171 | 43 854 |
| I45 | Other conduction disorders | 54 669 | 52 178 | 48 642 |
| I46 | Cardiac arrest | 12 304 | 11 260 | 10 767 |
| I47 | Paroxysmal tachycardia | 106 073 | 103 624 | 108 227 |
| I48 | Atrial fibrillation and flutter | 368 068 | 380 464 | 401 434 |
| I49 | Other cardiac arrhythmias | 388 871 | 419 048 | 435 380 |
| I50 | Heart failure | 432 681 | 454 187 | 524 217 |
| I51 | Complications and ill-defined descriptions of heart disease | 31 069 | 34 205 | 31 562 |
| I52 | Other heart disorders in diseases classified elsewhere | 8 721 | 8 084 | 8 280 |
| I60 | Subarachnoid haemorrhage | 56 523 | 54 753 | 63 700 |
| I61 | Intracerebral haemorrhage | 54 146 | 51 410 | 51 611 |
| I62 | Other nontraumatic intracranial haemorrhage | 4 623 | 5 623 | 4 665 |
| I63 | Cerebral infarction | 313 618 | 340 271 | 368 794 |
| I64 | Stroke, not specified as haemorrhage or infarction | 34 354 | 32 998 | 31 416 |
| I65 | Occlusion and stenosis of precerebral arteries, not resulting in cerebral infarction | 35 236 | 39 259 | 37 363 |
| I66 | Occlusion and stenosis of cerebral arteries, not resulting in cerebral infarction | 11 103 | 11 191 | 9 881 |
| I67 | Other cerebrovascular diseases | 100 801 | 108 145 | 113 758 |
| I68 | Cerebrovascular disorders in diseases classified elsewhere | 9 051 | 8 645 | 8 098 |
| I69 | Sequelae of cerebrovascular disease | 441 799 | 460 998 | 471 995 |
| I70 | Atherosclerosis | 279 683 | 278 841 | 279 814 |
| I71 | Aortic aneurysm and dissection | 92 036 | 97 564 | 108 498 |
| I72 | Other aneurysm and dissection | 30 883 | 35 853 | 39 144 |
| I73 | Other peripheral vascular diseases | 38 055 | 33 695 | 31 186 |
| I74 | Arterial embolism and thrombosis | 25 759 | 29 394 | 30 893 |
| I77 | Other disorders of arteries and arterioles | 17 926 | 18 120 | 19 566 |
| I78 | Diseases of capillaries | 5 291 | 5 657 | 5 978 |
| I79 | Disorders of arteries, arterioles and capillaries in diseases classified elsewhere | 4 215 | 4 400 | 4 039 |
| I80 | Phlebitis and thrombophlebitis | 538 385 | 550 399 | 533 491 |
| I81 | Portal vein thrombosis | 4 625 | 4 807 | 4 790 |
| I82 | Other venous embolism and thrombosis | 22 596 | 24 787 | 24 553 |
| I83 | Varicose veins of lower extremities | 1 272 990 | 1 340 925 | 1 275 990 |
| I84 | Haemorrhoids | 368 860 | 387 968 | 389 537 |
| I85 | Oesophageal varices | 12 868 | 12 909 | 13 516 |
| I86 | Varicose veins of other sites | 33 641 | 32 108 | 33 008 |
| I87 | Other disorders of veins | 98 311 | 101 106 | 100 749 |
| I88 | Nonspecific lymphadenitis | 7 907 | 10 231 | 8 651 |
| I89 | Other noninfective disorders of lymphatic vessels and lymph nodes | 20 329 | 22 408 | 20 930 |
| I95 | Hypotension | 13 429 | 12 457 | 11 605 |
| I97 | Postprocedural disorders of circulatory system, not elsewhere classified | 9 594 | 8 392 | 8 656 |
| I98 | Other disorders of circulatory system in diseases classified elsewhere | 10 598 | 10 298 | 10 614 |
| I99 | Other and unspecified disorders of circulatory system | 9 778 | 9 775 | 9 423 |
|  | **TOTAL** | **12 233 203** | **12 239 697** | **12 210 793** |

Source: Own elaboration based on CSO and SII data.

# **PRESENTEEISM**

Appendix 3. The total population with cardiovascular diseases in 2015-2017.

|  | 2015 | 2016 | 2017 |
| --- | --- | --- | --- |
| The total population with cardiovascular diseases | 1,160,667 | 1,160,667* | 1,160,667* |

*assumption

Source: <https://www.gov.pl/documents/292343/436711/polkard-2017-2020-aktualizacja+pdf.pdf/f9a56b63-9ad2-060d-40f1-f84ea520f3b8?fbclid=IwAR0iWqmtJJ__AlalaT87pvHIcGW-JvNIsI_LKWyE8LzScvPBntCV2i_7bDY> (page 10)

Appendix 4. Working-age with cardiovascular diseases in Poland in 2015-2017.

|  | 2015 | 2016 | 2017 |
| --- | --- | --- | --- |
| Working-age population with cardiovascular diseases | 447,807 | 461,854 | 474,455 |

Source: own elaboration.

# **PERMANENT AND TEMPORARY INCAPACITY FOR WORK (PENSION)**

Appendix 5. Number of certificates of permanent or temporary incapacity for work caused by cardiovascular diseases (ICD-10: I00-I99) in the 2015-2017 years in Poland.

|  | | 2015 | | | | 2016 | | | | 2017 | | | |
| --- | --- | --- | --- | --- | --- | --- | --- | --- | --- | --- | --- | --- | --- |
| ICD-10 | | Decisions determining the degree of incapacity for work | | | | Decisions determining the degree of incapacity for work | | | | Decisions determining the degree of incapacity for work | | | |
|  |  | 1) inability to live independently | 2) total incapacity for work | 3) partial incapacity for work | Total (1+2) | 1) inability to live independently | 2) total incapacity for work | 3) partial incapacity for work | Total (1+2) | 1) inability to live independently | 2) total incapacity for work | 3) partial incapacity for work | Total (1+2) |
| I00-I99 | Cardiovascular diseases | 4,940 | 12,879 | 34,169 | 17,819 | 4,382 | 10,998 | 28,298 | 15,380 | 4,303 | 11,094 | 29,142 | 15,397 |

Source: Own elaboration based on SII data.

Appendix 6. Expected number of years that a working age individual is able to work.

| Age | Expected number of years that a working age individual is able to work | Age | Expected number of years that a working age individual is able to work |
| --- | --- | --- | --- |
| 19 | 44.352 | 42 | 22.526 |
| 20 | 43.397 | 43 | 20.891 |
| 21 | 42.442 | 44 | 19.974 |
| 22 | 41.486 | 45 | 19.060 |
| 23 | 40.530 | 46 | 18.152 |
| 24 | 39.572 | 47 | 17.247 |
| 25 | 38.613 | 48 | 16.347 |
| 26 | 37.654 | 49 | 14.679 |
| 27 | 36.695 | 50 | 13.780 |
| 28 | 35.737 | 51 | 12.885 |
| 29 | 34.781 | 52 | 11.992 |
| 30 | 33.824 | 53 | 11.101 |
| 31 | 32.869 | 54 | 10.211 |
| 32 | 31.915 | 55 | 8.472 |
| 33 | 30.963 | 56 | 7.567 |
| 34 | 30.013 | 57 | 6.658 |
| 35 | 29.064 | 58 | 5.743 |
| 36 | 28.119 | 59 | 4.820 |
| 37 | 27.177 | 60 | 2.945 |
| 38 | 26.239 | 61 | 1.981 |
| 39 | 25.304 | 62 | 0.987 |
| 40 | 24.374 | 63* | 0.493 |
| 41 | 23.448 | 64* | 0.492 |
|  |  | 65* | 0.491 |

*Age 63-65, the formula (10) takes into consideration the coefficient ½ with respect to the fact that only men work at that age; the additional coefficient ½ has been taken into consideration for the age of 65 with respect to the statistical likelihood of working only six months because of retirement in that year. The consequences of the reform increasing the retirement age have not been taken into account.

Source: Own elaboration based on CSO data.

Appendix 7. The proportion of the age group in the total number of people with a certificate of permanent or temporary incapacity for work.

| Age | The proportion of the age group | Product of proportion and expected number of future years of work | Age | The proportion of the age group | Product of proportion and expected number of future years of work |
| --- | --- | --- | --- | --- | --- |
| 19 | 0.001473353 | 0.065346446 | 55 | 0.063455774 | 0.537571301 |
| 20 | 0.001778184 | 0.077167612 | 56 | 0.064217853 | 0.485948695 |
| 21 | 0.001981405 | 0.084094684 | 57 | 0.066554895 | 0.443144455 |
| 22 | 0.002489458 | 0.103278871 | 58 | 0.062947721 | 0.361527019 |
| 23 | 0.003149926 | 0.127665286 | 59 | 0.047553727 | 0.229213242 |
| 24 | 0.003454758 | 0.136710816 | 60 | 0.040796627 | 0.12013505 |
| 25 | 0.003962811 | 0.153015647 | 61 | 0.033125032 | 0.065609094 |
| 26 | 0.006249047 | 0.235301068 | 62 | 0.030229132 | 0.029835548 |
| 27 | 0.006807905 | 0.249816834 | 63 | 0.016968958 | 0.008364339 |
| 28 | 0.00762079 | 0.272345908 | 64 | 0.006553879 | 0.003226606 |
| 29 | 0.007519179 | 0.261521181 | 65 | 0.009856221 | 0.002423083 |
| 30 | 0.008840116 | 0.299009934 |  |  |  |
| 31 | 0.007671595 | 0.25215926 |  |  |  |
| 32 | 0.010669105 | 0.340508871 |  |  |  |
| 33 | 0.008941726 | 0.276862763 |  |  |  |
| 34 | 0.009703805 | 0.291235554 |  |  |  |
| 35 | 0.009653 | 0.280558172 |  |  |  |
| 36 | 0.009754611 | 0.274292041 |  |  |  |
| 37 | 0.010313468 | 0.28029037 |  |  |  |
| 38 | 0.010923132 | 0.286607682 |  |  |  |
| 39 | 0.012955342 | 0.327823533 |  |  |  |
| 40 | 0.012650511 | 0.308340762 |  |  |  |
| 41 | 0.01341259 | 0.314493303 |  |  |  |
| 42 | 0.014428695 | 0.325015296 |  |  |  |
| 43 | 0.015292384 | 0.319466624 |  |  |  |
| 44 | 0.015139969 | 0.302398312 |  |  |  |
| 45 | 0.018543921 | 0.353455482 |  |  |  |
| 46 | 0.018899558 | 0.343058162 |  |  |  |
| 47 | 0.023421226 | 0.403946595 |  |  |  |
| 48 | 0.023675253 | 0.387013438 |  |  |  |
| 49 | 0.028298532 | 0.415386224 |  |  |  |
| 50 | 0.033328253 | 0.459272322 |  |  |  |
| 51 | 0.036935427 | 0.47590743 |  |  |  |
| 52 | 0.046588427 | 0.55869307 |  |  |  |
| 53 | 0.053345527 | 0.592191891 |  |  |  |
| 54 | 0.057867195 | 0.590866304 |  |  |  |

Source: Own elaboration based on CSO data.

# **MORTALITY**

Appendix 8. Expected number of years that a working age individual is able to work together with a defined average number of lost working years in result of death by the age of the deceased (based on survival tables for Poland).

| Age | Expected number of years that a working age individual is able to work | Average number of lost working years in result of death | Age | Expected number of years that a working age individual is able to work | Average number of lost working years in result of death |
| --- | --- | --- | --- | --- | --- |
| 20 | 43.397 | 41.5 | 45 | 19.060 | 17.1 |
| 21 | 42.442 |  | 46 | 18.152 |  |
| 22 | 41.486 |  | 47 | 17.247 |  |
| 23 | 40.530 |  | 48 | 16.347 |  |
| 24 | 39.572 |  | 49 | 14.679 |  |
| 25 | 38.613 | 36.7 | 50 | 13.780 | 12.0 |
| 26 | 37.654 |  | 51 | 12.885 |  |
| 27 | 36.695 |  | 52 | 11.992 |  |
| 28 | 35.737 |  | 53 | 11.101 |  |
| 29 | 34.781 |  | 54 | 10.211 |  |
| 30 | 33.824 | 32.4 | 55 | 8.472 | 6.7 |
| 31 | 32.869 |  | 56 | 7.567 |  |
| 32 | 31.915 |  | 57 | 6.658 |  |
| 33 | 30.963 |  | 58 | 5.743 |  |
| 34 | 30.013 |  | 59 | 4.820 |  |
| 35 | 29.064 | 27.2 | 60 | 2.945 | 1.2 |
| 36 | 28.119 |  | 61 | 1.981 |  |
| 37 | 27.177 |  | 62 | 0.987 |  |
| 38 | 26.239 |  | 63* | 0.493 |  |
| 39 | 25.304 |  | 64* | 0.492 |  |
| 40 | 24.374 | 22.2 | 65* | 0.491 |  |
| 41 | 23.448 |  |  | | |
| 42 | 22.526 |  |  |  |  |
| 43 | 20.891 |  |  |  |  |
| 44 | 19.974 |  |  |  |  |

*Age 63-65, the formula (10) takes into consideration the coefficient ½ with respect to the fact that only men work at that age; the additional coefficient ½ has been taken into consideration for the age of 65 with respect to the statistical likelihood of working only six months because of retirement in that year. The consequences of the reform increasing the retirement age have not been taken into account.

Source: Own elaboration based on CSO data.

Appendix 9. Deaths caused by cardiovascular diseases (ICD-10: I00-I99), 2015-2017 in Poland.

| ICD-10  I00-I99 | The deceased’s age | | | | | | | | |
| --- | --- | --- | --- | --- | --- | --- | --- | --- | --- |
|  | 20-24 | 25-29 | 30-34 | 35-39 | 40-44 | 45-49 | 50-54 | 55-59 | 60-65 |
| 2015 | 87 | 184 | 377 | 697 | 1 195 | 2 152 | 3 954 | 7 809 | 11 963 |
| 2016 | 63 | 145 | 310 | 530 | 1 036 | 1 864 | 3 325 | 6 736 | 10 966 |
| 2017 | 62 | 111 | 276 | 487 | 871 | 1 536 | 2 749 | 5 868 | 10 051 |

Source: Own elaboration based on CSO data.
